# Experimental and Theoretical Investigation of Gadolinium Oxyhydride (GdHO) Thin Films: Optical, Photocatalytic, and Electronic Properties

**DOI:** 10.3390/nano13243093

**Published:** 2023-12-07

**Authors:** Kasi Vinoth Kumar, Luminita Andronic, Elbruz Murat Baba, Dargie Deribew, Jeyanthinath Mayandi, Ellen Moons, Smagul Zh. Karazhanov

**Affiliations:** 1 Department for Solar Energy, Institute for Energy Technology, 2027 Kjeller, Norway; vinothphysica@gmail.com (K.V.K.); elbruz.murat.baba@ife.no (E.M.B.); smagul.karazhanov@ife.no (S.Z.K.); 2 School of Physics, Madurai Kamaraj University, Madurai 625021, Tamil Nadu, India; 3 Department of Product Design, Mechatronics and Environment, Transilvania University of Brasov, 500036 Brasov, Romania; 4 Department of Engineering and Physics, Karlstad University, SE-65188 Karlstad, Sweden; deribew@kau.se (D.D.); ellen.moons@kau.se (E.M.); 5 School of Chemistry, Madurai Kamaraj University, Madurai 625021, Tamil Nadu, India; mayandi@gmail.com

**Keywords:** gadolinium oxyhydride (GdHO), photochromic properties, photocatalytic activity, work function, density functional theory (DFT)

## Abstract

Oxyhydrides of rare-earth metals (REMOHs) exhibit notable photochromic behaviors. Among these, yttrium oxyhydride (YHO) stands out for its impressive transparency and swift UV-responsive color change, positioning it as an optimal material for self-cleaning window applications. Although semiconductor photocatalysis holds potential solutions for critical environmental issues, optimizing the photocatalytic efficacy of photochromic substances has not been adequately addressed. This research advances the study of REMOHs, focusing on the properties of gadolinium oxyhydride (GdHO) both theoretically and experimentally. The electronic and structural characteristics of GdHO, vital for ceramic technology, are thoroughly examined. Explicitly determined work functions for GdH_2_, GdHO, and Gd_2_O_3_ stand at 3.4 eV, 3.0 eV, and 4.3 eV, respectively. Bader charge analysis showcases GdHO’s intricate bonding attributes, whereas its electron localization function majorly presents an ionic nature. The charge neutrality level is situated about 0.33 eV below the top valence band, highlighting these materials’ inclination for acceptor-dominant electrical conductivity. Remarkably, this research unveils GdHO films’ photocatalytic capabilities for the first time. Even with their restricted surface due to thinness, these films follow the Langmuir–Hinshelwood degradation kinetics, ensuring total degradation of methylene blue in a day. It was observed that GdHO’s work function diminishes with reduced deposition pressure, and UV exposure further decreases it by 0.2 eV—a change that reverts post-UV exposure. The persistent stability of GdHO films, hinting at feasible recyclability, enhances their potential efficiency, underlining their viability in practical applications. Overall, this study accentuates GdHO’s pivotal role in electronics and photocatalysis, representing a landmark advancement in the domain.

## 1. Introduction

Oxyhydrides of rare-earth metals (REMOHs) represent a class of substances that exhibit photochromic characteristics under normal conditions [1,2]. The unique properties and versatile applications of rare earth metals, including lanthanum, cerium, yttrium, and neodymium, in combination with their compatibility with thin-film technologies, make them compelling candidates for advanced materials research. Among REMOHs, yttrium oxyhydride (YHO) is the most extensively studied. These films, transparent to visible light with average transmittance exceeding 80%, can decrease their transmittance to ~20–50% upon UV illumination within ~10 min, a reversible color change [3]. Films with a lower oxygen concentration (δ ≤ 0.5, where δ is an oxygen-to-yttrium ratio of YH_2−δ_O_δ_) possess a bandgap of ≈2.8 eV, absorbing violet and blue light [4,5]. These properties make YHO interesting for application in building windows. The technology readiness level of YHO-based technology is seven, nearing commercialization. YHO also exhibits photocatalytic and self-cleaning properties [6], adding value to its use in window applications. However, other rare earth metal oxyhydrides besides YHO have not been studied extensively. While studies have focused on photochromism and related properties, other functionalities have received less attention.

Population growth and industrial development have led to environmental challenges, like air and water pollution. Semiconductor photocatalysis holds promise for addressing these issues [7]. Photocatalysis is a process in which a semiconductor exposed to light generates an electron–hole pair [8], and a redox reaction occurs on the catalyst surface [9]. Titanium oxide (TiO_2_) is the most widely studied photocatalyst due to its abundance, low toxicity, and high chemical stability [10,11]. Its major drawback is a wide bandgap of 3.2 eV, rendering it insensitive to visible light [12]. Numerous studies aim to improve TiO_2_ performance yearly by shifting the absorption edge [13,14]. Conversely, enhancing the photocatalytic performance of photochromic materials to increase visible light absorption under illumination remains scarcely explored.

Work function is a critical parameter in evaluating the photocatalytic performance of a material. Such studies are available only for YH_3−2x_O_x_ before and after UV illumination [6,15]. No theoretical or experimental study exists for other REMOHs. 

The first systematic studies of REMOHs using first-principle calculations have been performed [16,17,18,19]. Understanding film composition and establishing YHO’s chemical formula were crucial steps.

The theoretical model postulates that two hydride anions are replaced by one oxide anion, resulting in a chemical composition of YH_3−2x_O_x_ with x = 0.25 [16]. This model aligns well with the experimental data [20]. The theoretical predictions have been experimentally confirmed [21], and other chemical formulas, such as YH_2-x_O_x_, have been suggested [2]. The challenges of finding the unit cells of YH_3−2x_O_x_ due to the oxidation of the YH_2−δ_ lattice and plotting the phase portrait have been addressed [17]. Through ion beam analysis, these theoretical predictions have been experimentally confirmed Moldarev et al., 2018 [5]. The electronic properties and chemical bonding of YH_3−2x_O_x_ have been studied [3,19], but work on other REMOHs has not been systematic.

This study conducted a theoretical and experimental investigation on gadolinium oxyhydride to uncover new properties of the material. The primary objectives were to examine the work function of GdHO, both theoretically and experimentally, and to compare it with those of GdH_2_ and Gd_2_O_3_. Relationships were determined between the work function of GdHO and various deposition factors, including pressure, H_2_ flux, the thickness of the film, and UV light exposure. The photocatalytic performance of GdHO was evaluated for the degradation of methylene blue molecules following ISO 10678:2010 [22]. Further, the chemical bonding, charge neutrality level, and effective density of its states were investigated—parameters that have not been explored in the previous scientific literature. Through this exploration, we aim to contribute not only to the understanding of GdHO but also to the broader field of rare earth metal-based materials, emphasizing their unique advantages and potential applications.

## 2. Methods and Computational Description

### 2.1. Experimental Description

Thin films of GdHO were developed on glass platforms through a dual-stage process. This involved reactive sputtering of a metallic objective in an Ar-H_2_ combined environment using a Leybold Optics A550V7 (Buhlergroup, Uzwil, Switzerland), magnetron apparatus and subsequent exposure to oxidation in an air setting. Several deposition parameters, such as the H_2_ to Ar flow ratio, comprehensive deposition pressure, and the film’s thickness, were adjusted to investigate their influence on the photocatalytic attributes (refer to Table 1). The use of oscillating substrate holders ensured the films had a uniform chemical makeup throughout the deposition process. The selection of H_2_/Ar ratios of 25 and 35 is based on prior optimizations conducted in the laboratory. These ratios were chosen for their ability to yield films with the most favorable properties in terms of structural quality, photochromic properties, and photocatalytic efficiency.

Optical properties were determined using an Ocean Optics QE65000 spectrometer (Ocean Optics, Inc., Largo, FL, USA) equipped with an integrated sphere, deuterium, and tungsten–halogen probe lights. The optical bandgap was extrapolated from the Tauc plot, assuming a direct transition [23]. The evaluation of photochromic properties involved illuminating the samples for half an hour with a collimated laser diode module (λ = 405 nm, W ≈ 4.5 mW), followed by the computation of the mean change in transmittance.

The contact potential difference (CPD) of GdHO samples was ascertained via the Kelvin probe technique using an Ag probe. These thin films were laid on ITO-coated glass for measurement. The work function was inferred from the CPD for highly oriented pyrolytic graphite (HOPG), presuming a work function of 4.6 eV [24].

The GdHO samples’ photocatalytic activity was assessed by monitoring the degradation of methylene blue (MB) in an aqueous solution under simulated VIS irradiation (25% UV and 75% VIS without IR). Thin films were irradiated under UV (peak wavelength of 365 nm) for 30 min in air before immersion in the MB solution. Films with a 2.5 × 2.5 cm^2^ surface were immersed in 50 mL of an MB aqueous solution with an initial 4 mg/L concentration. Photocatalytic experiments, performed at room temperature for up to 6 h, used an annular photocatalytic reactor with four UV TL-D 18W BLB Blacklight Blue Philips lamps (Philips, Hamburg, Germany) (low-pressure mercury vapor fluorescent lamps, with a peak wavelength of around 365 nm), and twelve visible Master TL-D Super 80 18W/865 Philips lamps (fluorescent lamp, 740 lm). The residual MB’s absorption spectrum was periodically recorded between 200 and 800 nm by sampling an aliquot of the MB solution. The degradation efficiency (η) of the MB was calculated with Equation (1).

(1) $$\eta =\frac{C_{0}-C_{t}}{C_{0}}\cdot 100,\;\%$$

where C_0_ and C_t_ represent the initial concentration of the MB after a given time interval, respectively. The degradation efficiency of the MB without a photocatalyst was less than 1% after 6 h of irradiation.

The MB photodegradation followed a first-order Langmuir–Hinshelwood kinetics model [25], as described by Equation (2) (applicable for low concentrations), and fit the experimental data for all experiments very well.

(2) $$\mathrm{lnC}={\mathrm{lnC}}_{0}-k_{\mathrm{app}}\cdot t$$

where C_0_ represents the initial MB concentration, C is the MB concentration at time t (minutes), and k_app_ is the apparent rate constant (h^−1^). The k_app_ is the slope of the graph lnC as a function of t, and R^2^ represents the correlation coefficient.

### 2.2. Computational Details, Methods, and Modeling

In this study, density functional theory (DFT) computations were conducted using the Vienna ab initio simulation package (VASP). [26,27] with the Perdew–Burke–Ernzerhof (PBE) generalized gradient approximation [10] and projector-augmented wave (PAW) pseudopotentials [28,29,30]. The kinetic energy cut-off was set at 520 eV, and the convergence conditions for the total energy were established at less than 10^−6^ eV. The force convergence requirements during geometry optimization were set at 0.001 eV/Å.

For the Brillouin zone sampling, a 9 × 9 × 9 k-points Monkhorst–Pack scheme was utilized [31]. Significantly, we employed Grimme’s DFT-D3 scheme (Damme, Germany) for dispersion corrections to account for van der Waals (vdW) interactions, critical for accurately modeling the long-range behavior of the system. This correction involves adjusting the dispersion coefficients based on the atomic geometry and a damping function [32,33], a process essential for ensuring the accuracy and realism of our computational results. In demonstrating the impact of this correction, Supplementary Table S1 presents a comparison of the work function values calculated with and without the DFT-D3 correction. This comparison underscores the importance of incorporating the DFT-D3 correction in accurately predicting the physical properties of our system, particularly in the context of vdW interactions.

Further details of our computational approach, including the use of VESTA-3 for structural visualization, are provided in this section [34]. The work function was calculated for a slab with a vacuum on both sides of the surface [35,36]. Theoretical calculations, such as the work function, Bader charge, charge density, the electron localization function (ELF), and electronic properties were carried out to study the interaction of GdHO system. The work function is described as the minimum energy necessary for an electron to move from the surface of a solid into the vacuum, as outline in Equation (3), in which E_vac_ represents the electrostatic potential near the vacuum region at the surface, while E_f_ is the Fermi energy of the slab [35,37,38].

Φ = E_vac_ − E_f_ (3)

The ELF is a measure of the probability of finding electron pairs with parallel spin (Hartree–Fock approximation) [39].

(4) $$\mathrm{ELF}=\frac{1}{1+\chi_{\sigma }^{2}\left(r\right)}$$

In simpler terms, the ELF indicates the likelihood of encountering another electron in proximity to a reference electron, assuming both have the same spin, as articulated in Equation (4). The term $\chi_{\sigma }^{2}\left(r\right)$ functions as a dimensionless index for localizing electrons within a homogeneous electron gas. The charge neutral level (CNL) is determined by equating the real-space Green’s function (G) to zero, as detailed in Equation (5). The density of states (DOS), denoted as N(E’), is inferred from the band structure analysis [40]. The integration process over the Brillouin zone (BZ) was performed without adjusting the bandgap.

(5) $$G\left(E\right)=\int_{\mathrm{BZ}}\int_{-\infty }^{\infty }\frac{N(E^{\prime })d\;E^{\prime }}{E-\;E^{\prime }}=0$$

## 3. Results and Discussion

### 3.1. Experimental Results and Discussions

**Figure 1:**
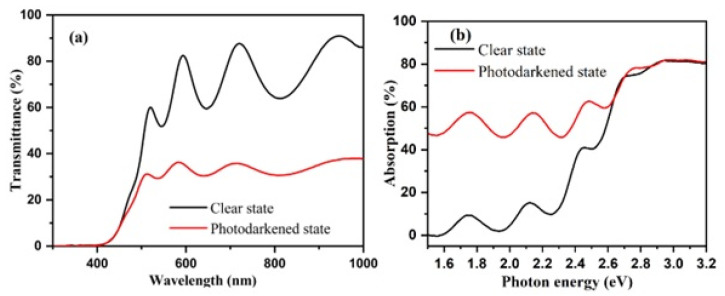
Spectral (**a**) transmission and (**b**) absorption of sample G2 in transparent and photodarkened states.

Figure 1a depicts the spectral transmissions of sample G2 in both its transparent and photodarkened states. The films exhibit a transparency of approximately 70% within the visible spectrum. However, after approximately 30 min of illumination, transmission decreases uniformly across the visible spectrum, while light absorption increases (as shown in Figure 1b). These changes in the optical properties are of significant interest, as they provide insights into the photochromic behavior of the material under investigation. The bandgap elevates from 2.8 eV to 3.1 eV as the deposition pressure ascends from 1.0 to 2.8 Pa. Prior research has established that REMOH films, synthesized at higher H_2_ pressure, contain an increased quantity of oxygen, likely due to enhanced porosity facilitating faster and more significant oxidation, and thus, resulting in a more significant bandgap [11,12,41]. Changes in the hydrogen flow and film thickness generate varying photochromic responses due to the resultant modification in the bandgap. The bandgap and the photochromic response after 30 min of illumination for each sample are summarized in Table 1.

**Figure 2:**
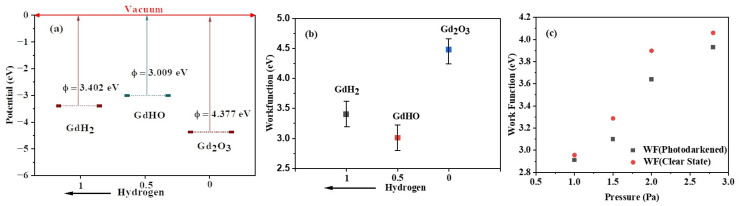
Theoretical vacuum potential (**a**) and work function (**b**) at varying hydrogen compositions. (**c**) Work function of the samples prepared under different deposition conditions determined by the Kelvin probe measurement.

When a material’s Fermi level is sufficiently high, photo-induced charge carriers can be transferred to the surface and react with an adsorbate. Therefore, managing the work function of REMOHs becomes notably significant. The work function of GdHO films was deduced from measurements taken by a Kelvin probe (see Figure 2a).

An increase in the work function was observed from 2.96 eV to 4.06 eV, corresponding with heightened deposition pressure and hydrogen flow. This modulation, spanning an energy range of about 1 eV, was ascribed to variances in the chemical composition. For instance, films deposited under increased pressure exhibited higher oxygen content and a lower hydrogen concentration [42]. Given that oxygen exhibits greater electronegativity than hydrogen, a boost in the oxygen concentration led to an amplified negative surface charge, resulting in a higher work function. Moreover, the work function is directly affected by the size and orientation of the crystal [43]. As noted by Baba et al. [44], GdHO films produced at lower deposition pressures tend to expose the (200) planes predominantly. A rise in the deposition pressure led to films with random orientations and smaller grain sizes. Previous studies have shown that a film’s texture and grain size also rely on its thickness [45]. This could account for the lower work functions observed in thinner films than in thicker ones.

Kelvin probe measurements were conducted on the film to investigate how the photochromic reaction impacts the work function in the transparent state and during bleaching after 20 min of photodarkening (see Figure 2c). Illumination led to a reduction in the work function by 0.2 eV, which was followed by a recovery when the light was turned off. In a similar context, Mongstad et al. [15] examined comparable YHO films and attributed the work function’s decline to the movement of the Fermi level towards the conduction band due to photo-induced excess carriers. Consequently, the restoration of the work function was linked with the recombination of these charge carriers, typically occurring within the microsecond to millisecond range.

Theoretical work function values for GdH_2_, GdHO, and Gd_2_O_3_ were calculated as 3.009 eV, 3.402 eV, and 4.337 eV, respectively, by aligning the Fermi level relative to the vacuum energy level (Figure 2a,b) [35,38].

**Figure 3:**
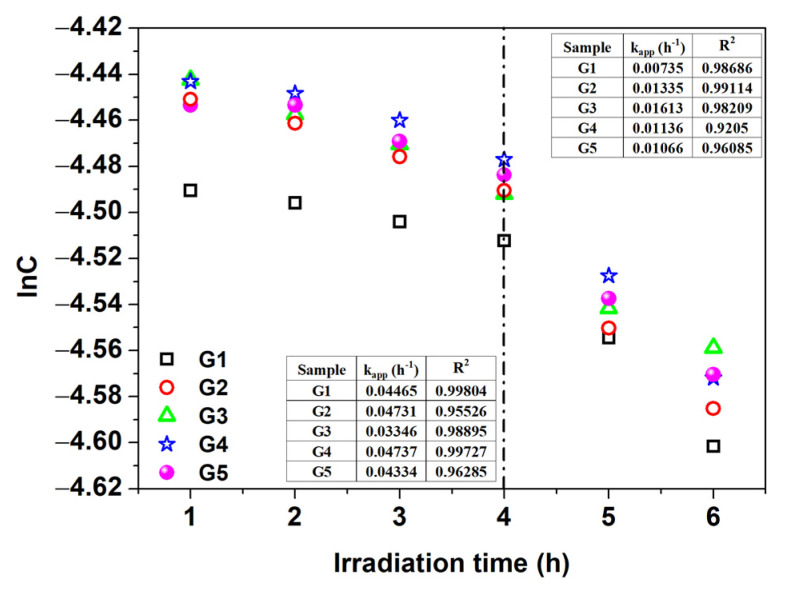
First-order Langmuir–Hinshelwood kinetics plot showing the apparent rate constants (k_app_) and correlation coefficients (R^2^) for the degradation of methylene blue (MB).

Photocatalytic properties of the REMOH films were investigated by analyzing methylene blue (MB) degradation kinetics in the presence of the REMOH surfaces. Before the photocatalytic degradation experiments started, 30 min in darkness was established to ensure the adsorption-desorption equilibrium of methylene blue (MB) on the REMOH film surfaces. This step is critical to ascertain that the subsequent kinetics measured are solely attributable to photocatalytic activity under irradiation and not influenced by surface adsorption effects. The experimental data exhibited excellent agreement with the Langmuir–Hinshelwood kinetic model (according to Equation (2)), as confirmed by a correlation coefficient exceeding 0.97. Two distinct kinetic behaviors were observed for all catalysts: an initial slower phase during the first 4 h of photocatalysis under UV irradiation and a faster kinetics phase after that. The acceleration in the second phase can be attributed to the generation of hydroxyl radicals on the photocatalyst surface, which play a crucial role in driving the photodegradation process (as illustrated in Figure 3). Beyond 4 h of UV-VIS irradiation, the degradation process displayed a linear pattern. Based on the reaction kinetics, it is hypothesized that approximately 50% of the MB will be degraded within 12 h, with complete photodegradation anticipated within 24 h. Moreover, the work function of GdHO films showcased a range from 3.2 eV to 4.1 eV, varying with the deposition pressure. This attribute can be further diminished by producing films under reduced pressures, leading to a lower concentration of oxygen. Despite the differences in the bandgap and work function, all samples initially showed minimal MB degradation during the first 4 h, which can be credited to substrate activation. Yet, efficiency displayed straight-line growth following 4 h of exposure to light (refer to Figure 3).

### 3.2. Theoretical Insights into the GdHO

Two distinct stable crystal configurations, as illustrated in Supplementary Figure S1a,b, corresponding to the GdHO adopting cubic P-43m (No. 215) and cubic F-43m (No. 216) structures in Supplementary Figure S1, were studied. The theoretical lattice parameters and bandgap values for these configurations are detailed in Supplementary Table S2. Notably, GdHO (No. 215) exhibited a metallic character, whereas GdHO (No. 216) demonstrated semiconductor properties with a bandgap of 3.1 eV. The crystal model of GdHO was selected with the following parameters: lattice parameters *a* = *b* = *c* = 5.3658 Å; *α* = *β* = *γ* = 90°, and the space group F-43m. In the crystal structure, each Gd atom is bonded to four oxygen and four hydrogen atoms, resulting in a unit volume of 154.49 Å^3^. Theoretical calculated atomic Wyckoff positions and lattice constants for GdH_2_, GdHO, and Gd_2_O_3_ are summarized in Table 2.

In calculations utilizing the PBE and HSE06 methods, the bandgap obtained with HSE06 (alpha = 0.2) is overestimated by approximately 4.3 eV compared to the PBE method, as indicated in Supplementary Table S3 and Figure S2a. This discrepancy is nearly 1.2 eV greater than the bandgap calculated within the PBE. However, the experimentally observed optical bandgap falls within the range of 2.8 eV to 3.1 eV, as shown in Table 1. Consequently, we opted for the PBE method over HSE06 due to its better agreement with the experimental findings. The application of the GGA + U methods was crucial for this study, by varying the on-site Coulomb repulsion parameter U for the strongly correlated f electrons of Gd in the GdHO (No. 216) crystal system from 1 eV to 4 eV. This was accomplished using the simplified (rotationally invariant) approach introduced by Dudarev [46]. The theoretical lattice parameters, density, bandgap, ground state energy, and total density of states for the GdHO (No. 216) crystal system, obtained using the PBE(DFT) and PBE(DFT + U) methods, are detailed in Supplementary Table S3 and Figure S2b. Introducing the on-site interaction potential resulted in a reduction of the lattice constant to 5.14 Å and an increase in the system’s density to 8.5 g/cm^3^. However, the experimental lattice constant was observed to be 5.42 Å [5], consistent with the GGA approximation value of 5.36 Å. Consequently, the GGA approximation is favorable for the GdHO system.

**Figure 4:**
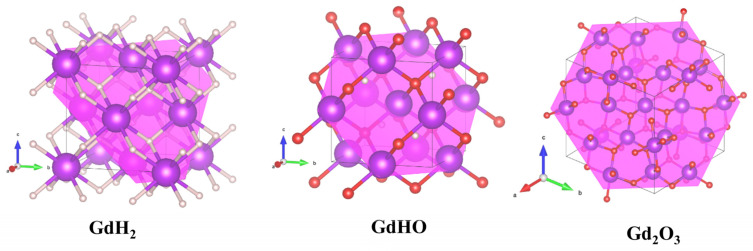
Unit cell representation of GdH_2_, GdHO, and Gd_2_O_3_, along with the cross-section along the (111) plane.

The density and lattice constant gradually Increase as the hydrogen content decreases and the oxygen content rises in the crystal system. Slabs were constructed based on optimized primitive cells, and the (111) plane was sliced for work function calculations. From the XRD pattern of GdH_2_, GdHO, and Gd_2_O_3_, the Miller indices predominantly align along the (111) and (200) planes, as shown in Supplementary Figure S3. Notably, both the experimental and theoretical data indicate that the (111) plane exhibited the intense peak, and the most stable configuration was chosen for the work function calculation [47,48]. Planar slabs were created for GdH_2_, GdHO, and Gd_2_O_3_, as depicted in Figure 4.

**Figure 5:**
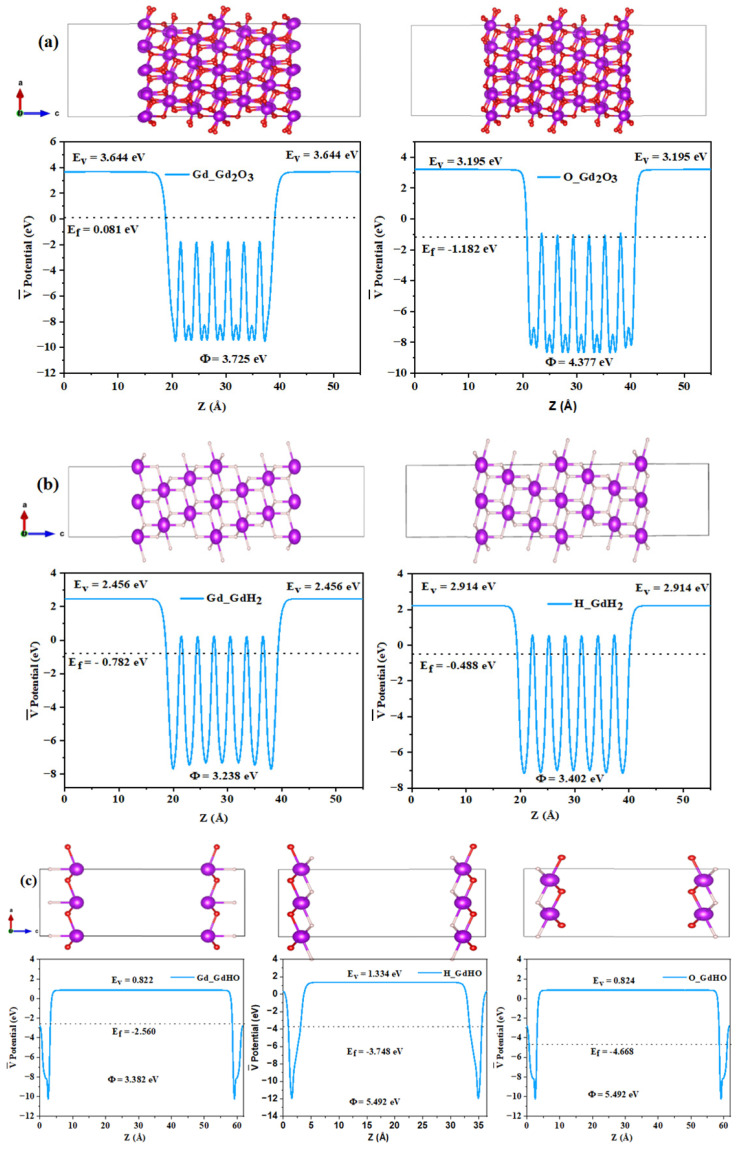
The work function of different surface terminations: (**a**) Gd- and O-terminated Gd_2_O_3_, (**b**) Gd- and H-terminated GdH_2_, and (**c**) Gd-, H-, and O-terminated GdHO surfaces.

The thickness of the constructed plane was set to 10 Å to accurately represent the properties of the thin film, as illustrated in Figure 5. Additionally, a surface vacuum layer, with a thickness of 40 Å, was included to minimize the electrostatic interactions between the layers along the Z-axis.

### 3.3. Computation of Work Function

In Figure 5, the work functions of different surface terminations in GdHO are depicted. The GdHO surfaces were terminated with H, Gd, and O, and their respective work function values were determined. For H-, Gd-, and O-terminated GdHO slabs, the Fermi energy sifts were observed at −1.781 eV, −2.560 eV, and −4.668 eV, respectively. Correspondingly, the vacuum level decreased from 1.228 eV to 0.882 eV and 0.824 eV for the Gd-terminated and O-terminated GdHO surfaces compared to the H-terminated surfaces. The surface work function exhibited an increase of 0.373 eV for the Gd-terminated and of 2.483 eV for the O-terminated GdHO surfaces. Similarly, the O-terminated Gd_2_O_3_ surface showed a higher work function of 0.652 eV compared to the Gd surface. However, the H-terminated GdH_2_ surface demonstrated a modest increase in work function by 0.164 eV. These findings suggest that the presence of oxygen atoms at the surface contributes to an elevation in the work function. Based on our theoretical results, the work function of GdH_2_, GdHO, and Gd_2_O_3_ collectively impacts the properties of the thin films. The work function is closely linked to the catalytic activation energy, which represents the energy barrier that must be overcome for a reaction to occur in the presence of the catalyst.

According to Guisbiers et al. [49], the most active catalyst exhibits the lowest activation energy. Catalytic activation energy is a critical kinetic parameter associated with chemical activity. Notably, the activation energy of the catalyst, linked to its work function, demonstrated a linear relationship. This suggests that the rate of the dye degradation reaction could be correlated with the work function of GdHO. As depicted in Figure 4, the degradation efficiency of methylene blue increases linearly with extended irradiation time, directly associated with the work function.

### 3.4. Chemical Bonding

**Figure 6:**
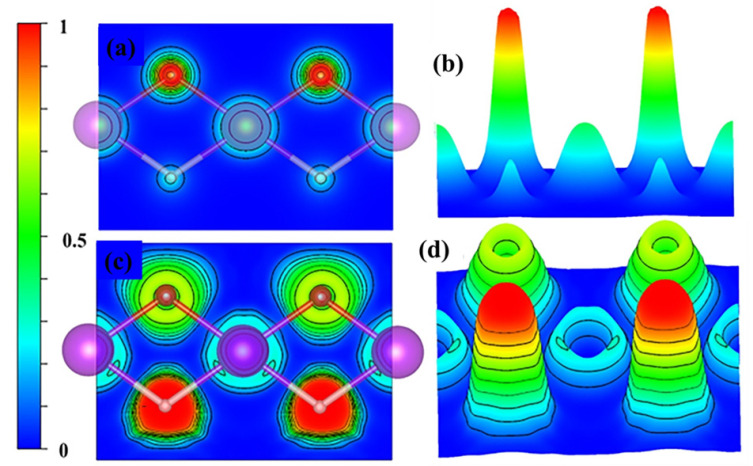
The 2- and 3D plots of the (**a**,**b**) charge density and (**c**,**d**) electron localization function (ELF) for GdHO.

**Figure 7:**
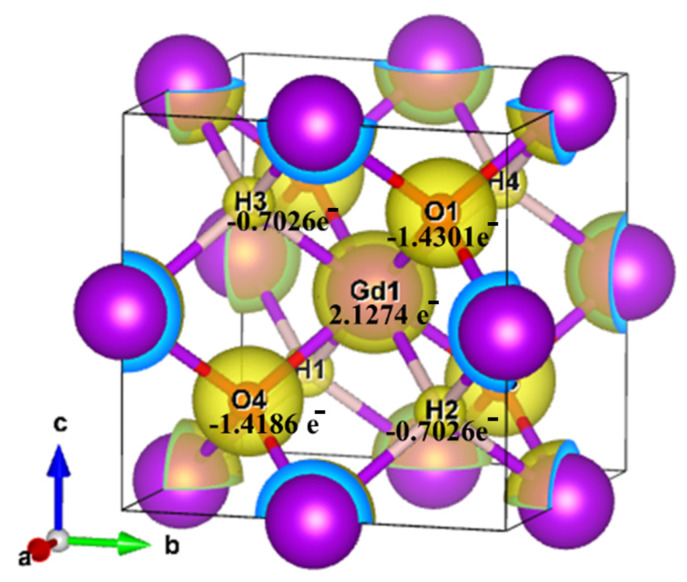
Bader charge of Bulk GdHO.

Electron localization function (ELF) is closely related to the electron density and shares several advantages with Bader analysis. In Figure 6a,b, the charge distribution among the Gd, O, and H atoms in the GdHO molecule is depicted. It can be observed that electrons are localized around the oxygen atoms due to their high electron affinity. The PAW method used in the computation considers only the valence electrons, which explains the zero value in the ELF profile at the core of the atoms (indicated by the blue circle in Figure 7c,d). The ELF values of approximately 1 and 0.5 for H and O, respectively, indicate the presence of an ionic bond in GdHO [50].

A grid-based Bader analysis algorithm [51] was utilized to analyze the charge distribution in bulk GdHO, as shown in Table 3. There was an observable accumulation of electrons around the O, and H atoms, while a depletion of electrons in the proximity of the Gd atom is revealed in the contour plot of the charge density of bulk GdHO (refer to Figure 7). As per the Bader charge analysis, Gd donated 2.12 e^−^ to the O and H atoms, which gained around −1.43 e^−^ and −0.70 e^−^, respectively, indicating an ionic nature of bonding. Consequently, the oxidation state of Gd is observed to be +3.

### 3.5. Electronic Properties

The density of states (DOS) of GdHO was computed to enhance theoretical insights into the material’s electronic characteristics. GdHO, identified as a wide bandgap semiconductor, exhibited a bandgap of ~ 2.8–3.1 eV under varying pressure conditions, an attribute achieved using magnetron sputtering. The theoretical observations closely align with the experimental results.

**Figure 8:**
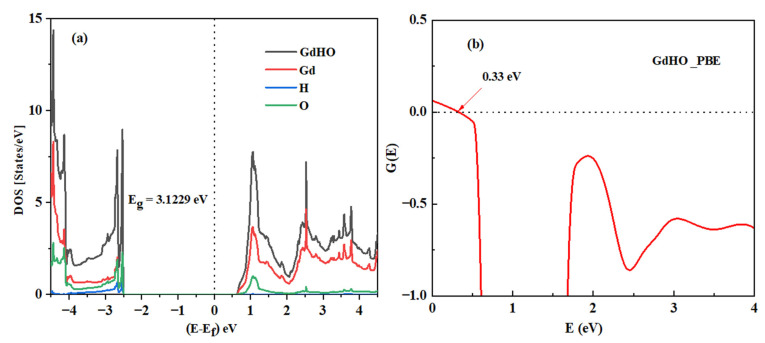
(**a**) Density of states (DOS) of GdHO and (**b**) representation of charge neutrality level in GdHO.

For the calculation of the density of state, the standard tetrahedron method, coupled with spin-polarized calculations, was implemented. The Fermi energy level was set to zero in these computations. As depicted in Figure 8a, the valence band maximum (VBM) predominantly consisted of oxygen’s (O) 2p orbital, with a partial overlap with gadolinium’s (Gd) 3d orbital, more than hydrogen’s (H) 1s at an energy level of −2.4954 eV. Conversely, the conduction band maximum (CBM) was dominated by the Gd 3d orbital at an energy of 0.6275 eV. These results verify that the 2p orbital of the GdHO oxide ion possessed higher energy than the hydride ion’s 1s orbital, corroborating a theoretical bandgap of 3.1229 eV. The CNL served as a pivotal criterion for identifying the energy level at which the crystal’s bandgap states transitioned from being donor-centric to acceptor-centric.

The CNL was calculated as a weighted average over the DOS. Consequently, the valence band (VB) and conduction band (CB) in the DOS competed to repel this energy level towards the midpoint of the bandgap. Similar to other p-type materials, GdHO exhibited a large, oxygen-like valence band with a high DOS, which strongly repelled the CNL towards the valence band maximum. As a result, the CNL was positioned 0.33 eV below the center of the bandgap for GdHO, as shown in Figure 8b. Therefore, undoped GdHO crystals predominantly exhibited p-type conductivity.

## 4. Conclusions

Gadolinium oxyhydride (GdHO)-based multi-anion thin films, both theoretically and experimentally studied, displayed remarkable photochromic properties under ambient conditions. When illuminated with UV light, the transmittance plummeted from ~70% to ~35% within approximately 10 min, returning to its original state once the illumination ceased. A variable work function, influenced by deposition parameters, emerged as a significant feature of these GdHO films. Specifically, a greater work function correlated with a higher deposition pressure, a phenomenon attributed to an increased oxygen concentration. While the hydrogen flow and film thickness influenced the work function only marginally, photodarkening reduced the work function by 0.2 eV, associated with the emergence of extra charge carriers. During the bleaching process, a gradual return of the contact potential difference (CPD) was observed.

From density functional theory (DFT) modeling, the electron localization function (ELF) of GdHO primarily indicates an ionic character influenced heavily by the electronegative oxygen. Concurrently, the Bader analysis determined the oxidation state of gadolinium (Gd) to be +3. Given the broad bandgap and a CNL of 0.33 eV, the feasibility of generating n-type materials through chemical impurity doping seems limited. Significantly, REMHO materials exhibited photocatalytic activity, especially after 4 h of light activation, positioning this material as an ideal candidate for environmentally beneficial window applications.

Our intensive examination of GdHO, juxtaposed with other rare-earth metal oxyhydrides like YHO, opens a new chapter in electronics and photocatalysis. The distinct photochromic behaviors, combined with its photocatalytic degradation properties of methylene blue, underscores GdHO’s immense potential, especially as a self-cleaning window solution.

Building upon these findings, the remarkable electronic and magnetic properties of GdHO films suggest their significant potential in advancing current technologies in electronics and heterogeneous catalysis. Additionally, the domain of energy storage and conversion also stands to benefit from the unique properties of GdHO films. Their robustness, coupled with a high work function, is particularly advantageous for the development of electrodes in batteries and fuel cells, offering prospects for improved energy density and stability. These attributes align well with the global initiative towards renewable energy sources, positioning GdHO films as a promising material for sustainable energy solutions.

## Figures and Tables

**Table 1:** Deposition parameters and resulting properties of GdHO samples.

Sample	Deposition Pressure (Pa)	H_2_:Ar (sccm)	Number of Passes	E_g_ (eV)	Photochromic Response (%)
G1	1.0	35:160	200	--	0
G2	1.5	35:160	200	2.8	39.37
G3	2.0	25:160	200	3.0	25.13
G4	2.0	35:160	200	3.0	24.15
G5	2.8	35:160	200	3.1	13.68

**Table 2:** Theoretical calculated lattice parameters of bulk GdH_2_, Gd_2_O_3_, and GdHO.

Chemical Formula	*a/b/c* (Å)	ρ (g/cm^3^)	Atom	Wyckoff Position	x	y	z
GdH_2_	5.2145	7.46	Gd	4a	0	0	0
			H	8c	¾	¾	¾
GdHO	5.3658	7.49	Gd	4b	½	½	½
			O	4d	¾	¾	¾
			H	4c	¼	¼	¼
Gd_2_O_3_	8.7948	9.19	Gd1	24d	½	¾	0.4676
			Gd2	8b	¾	¼	¼
			O	48e	0.8468	0.1220	0.3942

**Table 3:** Bader charge analysis and charge transfer in bulk GdHO.

Atom	Bader Charge (e^−^)	Charge Transfer (e^−^)
Gd_1_	6.87255	2.1274
O_1_	7.430157	−1.4301
H_1_	1.702618	−0.7026

## Data Availability

The data presented in this study are available on request from the corresponding author. The data are not publicly available due to privacy.

## Supplementary Materials

The supporting information can be downloaded at: https://www.mdpi.com/article/10.3390/nano13243093/s1.
