# Supplementary material for: Experimental and Theoretical Investigation of Gadolinium Oxyhydride (GdHO) Thin Films: Optical, Photocatalytic, and Electronic Properties
Source: Nanomaterials (Basel). 2023 Dec 7;13(24):3093. doi: 10.3390/nano13243093 (PMC10745946; doi:10.3390/nano13243093)
Supplement: Supplementary file 1 [file nanomaterials-13-03093-s001.zip › nanomaterials-2718215-supplementary.pdf]

nanomaterials

MDPI

Supplementary Information

Experimental and Theoretical Investigation of Gadolinium Oxyhydride (GdHO) Thin Films: Optical, Photocatalytic, and Electronic Properties

Kasi Vinoth Kumar [1,2], Luminita Andronic [3,*], Elbruz Murat Baba [1], Dargie Deribew [4], Jeyanthinath Mayandi [5], Ellen Moons [4] and Smagul Zh. Karazhanov [1]

[1] Department for Solar Energy, Institute for Energy Technology, 2027 Kjeller, Norway
[2] School of Physics, Madurai Kamaraj University, Madurai 625021, Tamil Nadu, India
[3] Department of Product Design, Mechatronics and Environment, Transilvania University of Brasov, Eroilor 29, 500036 Brasov, Romania
[4] Department of Engineering and Physics, Karlstad University, SE-65188 Karlstad, Sweden
[5] School of Chemistry, Madurai Kamaraj University, Madurai 625021, Tamil Nadu, India
* Correspondence: andronic-luminita@unitbv.ro

Table S1 Theoretical work function comparison with and without DFT- D3 correction for Gd, H, and O terminated GdHO surfaces.

System	With DFT- D3 correction			Without DFT- D3 correction		
	Work function(ϕ) (eV)	E_f (eV)	E_V (eV)	Work function(ϕ) (eV)	E_f (eV)	E_V (eV)
Gd_GdHO	3.38	-2.56	0.82	5.01	-2.92	2.08
H_GdHO	3.00	-1.78	1.22	5.02	-3.68	1.33
O_GdHO	5.49	-4.66	0.82	5.48	-4.66	0.82

Table S2 Theoretical lattice parameters and band gap for GdHO (No. 215) and GdHO (No. 216) crystal system.

Chemical formula	*a*/*b*/*c* [Å]	Crystal System and Space group	ϱ [g/cm³]	Atom	Wyckoff position	x	y	z	E_g (PBE) eV	E_g (HSE06) eV
GdHO (215)	5.39	CubicP-43m (no.215	7.36	Gd1	4e	0.23	0.23	0.23	-	0.4
				O1	1a	0	0	0		
				O1	3d	½	0	0		
				H1	3c	0	½	½		
				H1	1b	½	½	½		
GdHO (216)	5.36	CubicF-43m (no.216)	7.49	Gd	4b	½	½	½	3.1	4.3
				O	4d	¾	¾	¾		
				H	4c	¼	¼	¼		

Table S3 Theoretical lattice parameters, density, band gab and ground state energy for for GdHO (No. 216) crystal system using PBE(DFT), PBE(DFT+U) and HSE06 method.

Method GdHO (216)	*a/b/c* [Å]	P [g/cm³]	E_g (eV)	E_0 (eV)
PBE (DFT)	5.36	7.49	3.12	-115.33
(DFT+U) U= 1 eV	5.14	8.51	3.06	-113.70
U= 2 eV	5.12	8.57	3.16	-113.34
U= 3 eV	5.14	8.50	3.24	-113.31
U= 4 eV	5.14	8.50	3.30	-113.17
HSE06	5.36	7.49	4.30	-83.85

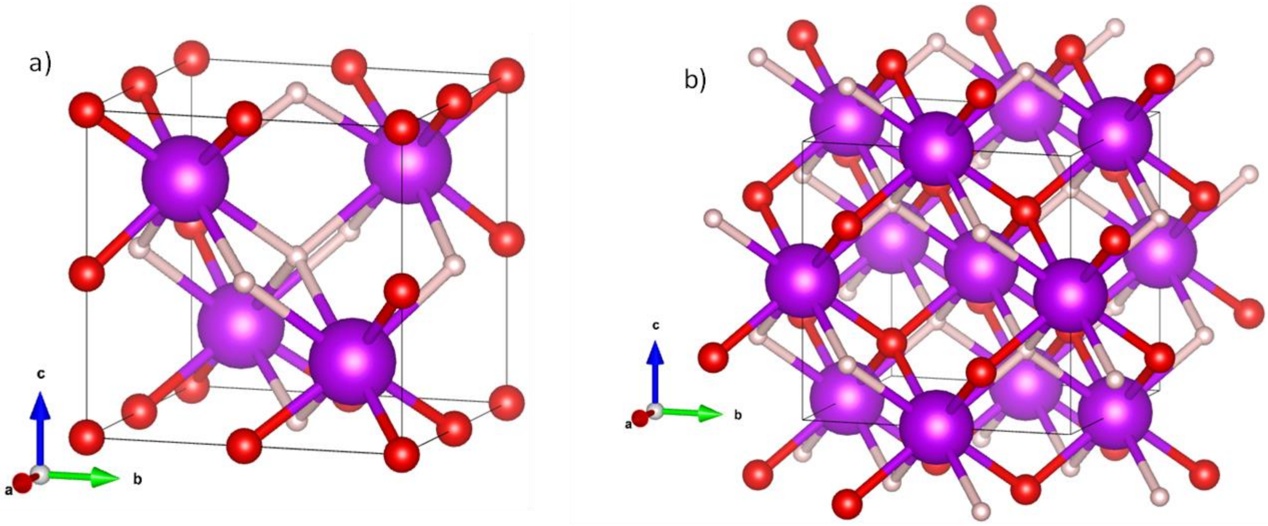

Figure S1 a) GdHO (No. 15) 2and b) GdHO (No. 216) crystal system.

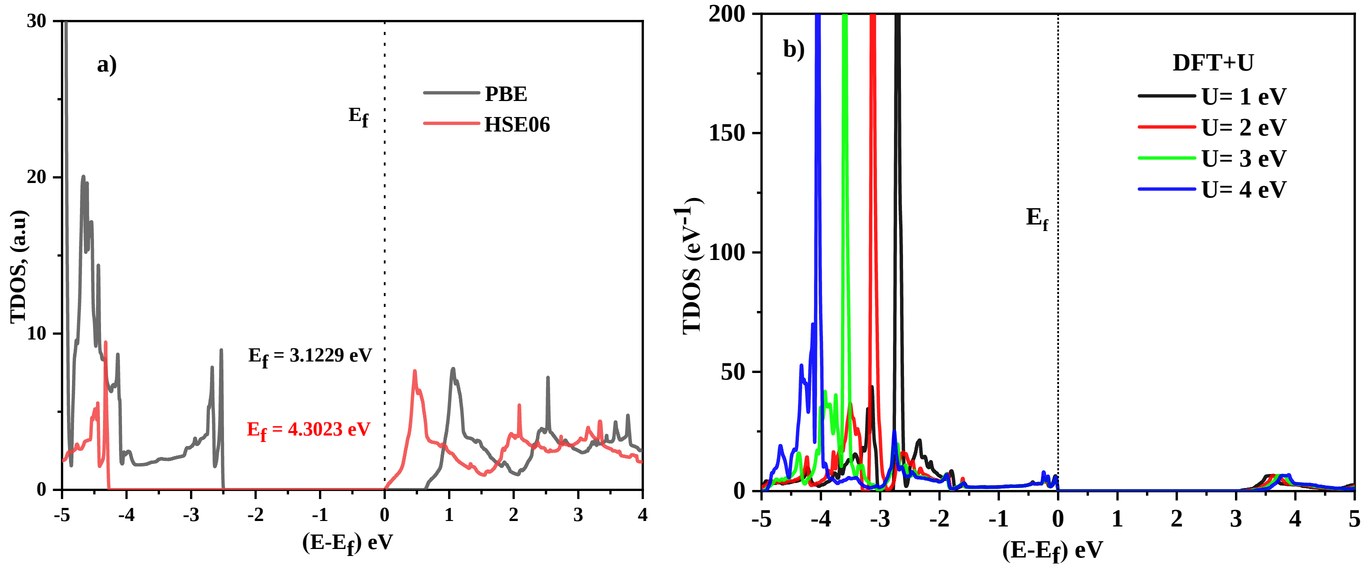

Figure S2 a) TDOS using PBE and HSE06 methods and b) TDOS using PBE (DFT+U) with varying on-site Coulomb repulsion (U).

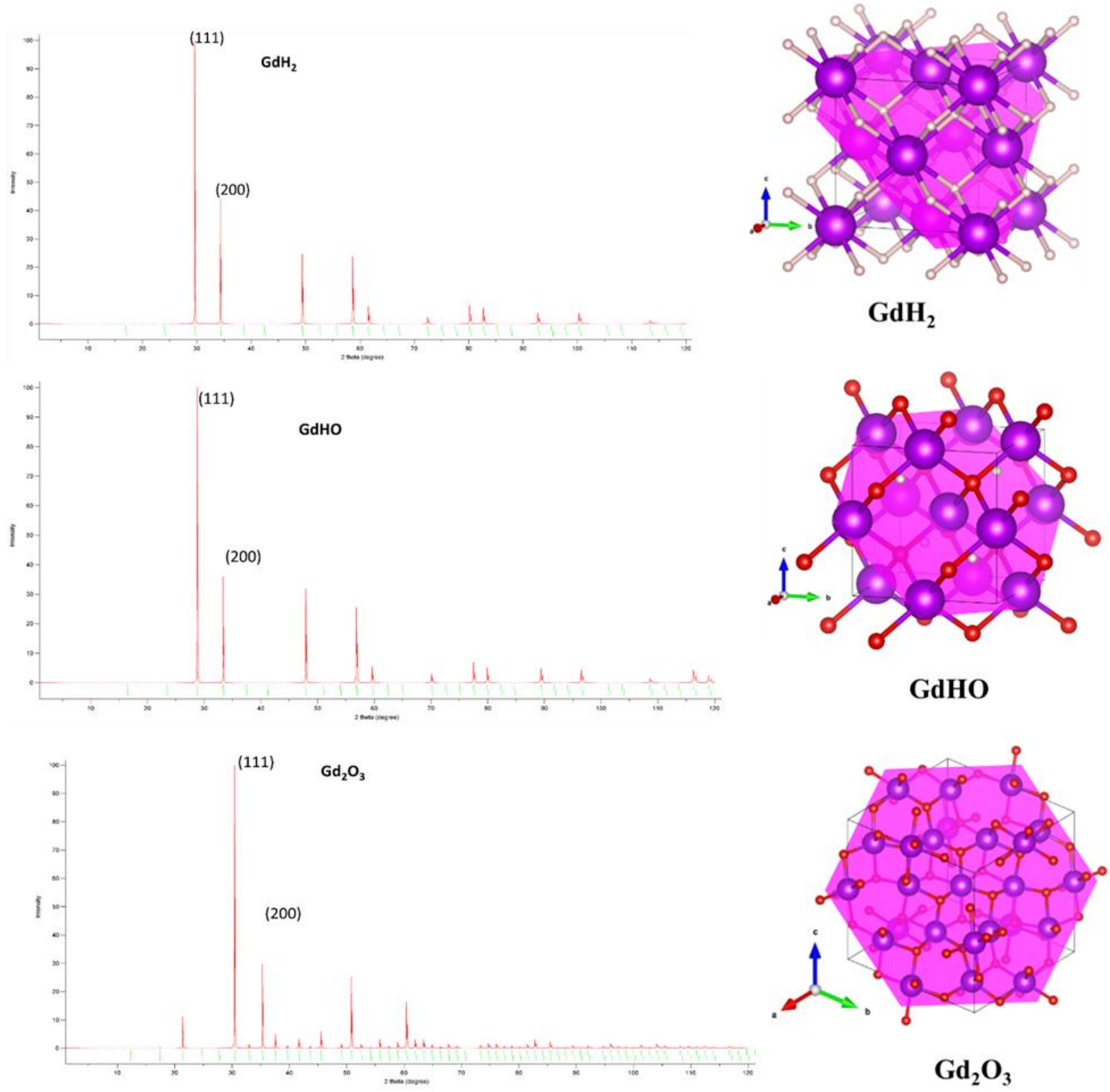

Figure S3 Theoretically obtained XRD pattern and Unit cell representation of GdH_2, GdHO, and Gd_2O_3 along with the cross-section along 111 plane.

Disclaimer/Publisher's Note: The statements, opinions and data contained in all publications are solely those of the individual author(s) and contributor(s) and not of MDPI and/or the editor(s). MDPI and/or the editor(s) disclaim responsibility for any injury to people or property resulting from any ideas, methods, instructions or products referred to in the content.
